# Robust Soft Sensor with Deep Kernel Learning for Quality Prediction in Rubber Mixing Processes

**DOI:** 10.3390/s20030695

**Published:** 2020-01-27

**Authors:** Shuihua Zheng, Kaixin Liu, Yili Xu, Hao Chen, Xuelei Zhang, Yi Liu

**Affiliations:** 1 Institute of Process Equipment and Control Engineering, Zhejiang University of Technology, Hangzhou 310023, China; zneu@zjut.edu.cn (S.Z.); kxliu@zjut.edu.cn (K.L.); 2 Shanghai Customs, Shanghai 200120, China; lily_xyl@163.com (Y.X.); zhangxueleippt@aliyun.com (X.Z.); 3 Quanzhou Institute of Equipment Manufacturing, Haixi Institutes, Chinese Academy of Sciences, Jinjiang 362200, China; chenhao@fjirsm.ac.cn

**Keywords:** soft sensor, deep learning, semi-supervised learning, robust estimator, ensemble strategy, rubber mixing process, Mooney viscosity

## Abstract

Although several data-driven soft sensors are available, online reliable prediction of the Mooney viscosity in industrial rubber mixing processes is still a challenging task. A robust semi-supervised soft sensor, called ensemble deep correntropy kernel regression (EDCKR), is proposed. It integrates the ensemble strategy, deep brief network (DBN), and correntropy kernel regression (CKR) into a unified soft sensing framework. The multilevel DBN-based unsupervised learning stage extracts useful information from all secondary variables. Sequentially, a supervised CKR model is built to explore the relationship between the extracted features and the Mooney viscosity values. Without cumbersome preprocessing steps, the negative effects of outliers are reduced using the CKR-based robust nonlinear estimator. With the help of ensemble strategy, more reliable prediction results are further obtained. An industrial case validates the practicality and reliability of EDCKR.

## 1. Introduction

The rubber mixing process is the first and important phase in tire and rubber manufacturing. During the process, natural rubber, synthetic raw materials, and additives are put into the internal mixer. After two to five minutes of mixing, the mixture is discharged to an extruder. In summary, the rubber mixing process is a complex nonlinear process performed in batches. The Mooney viscosity is one of the key quantities concerning end product quality. Despite the commercial importance, no comprehensive analysis of the rubber mixing process is currently available in practice. Additionally, the Mooney viscosity cannot be measured online, and instead it is only assayed offline in the lab with a large delay [1,2]. In such a situation, soft sensors (or inferential sensors) for quality modeling and prediction become very necessary in practice [3,4,5,6,7,8,9].

Current data-driven soft sensors for the Mooney viscosity information are generally divided into two categories, supervised and semi-supervised, according to the training datasets being labeled or semi-labeled. Most of the existing Mooney viscosity soft sensors belong to the first category, such as shallow neural networks (NNs) [10,11], partial least squares (PLS) [12,13], Gaussian process regression (GPR) [12,13,14,15], and extreme learning machine (ELM) [16]. Generally, they learn a labeled dataset $S^{l}=\left\{X^{l},Y\right\}$ with *N* pairs of input and output samples, denoted as $X^{l}={\left\{x_{i}^{l}\right\}}_{i=1}^{N}$ and $Y={\left\{y_{i}\right\}}_{i=1}^{N}$, respectively. One main disadvantage of these supervised prediction models is that the information hidden in *U* unlabeled samples (*U* >> *N*), denoted as $X^{u}={\left\{x_{i}^{u}\right\}}_{i=1}^{U}$, is omitted and not utilized. Alternatively, the semi-supervised soft sensors, such as semi-supervised ELM (SELM), enhance the prediction results (e.g., compared with ELM) by suitably modeling of both the labeled dataset $S^{l}$ and the unlabeled dataset $S^{u}=\left\{X^{u}\right\}$ [17]. To further improve the prediction accuracy, both supervised and semi-supervised soft sensors are further combined with the ensemble learning or just-in-time learning strategies in different scenarios [5,18,19,20].

For complex rubber mixing processes, without enough prior knowledge, the suitable selection and exaction of input variables is not easy. Although the traditional principal component analysis (PCA) and PLS preprocessing approaches can be used to extract latent variables, they are both linear [4]. Additionally, most PCA-related analysis methods process multivariate data in their raw forms. Alternatively, the representation of data at a deeper level reveals inherent features and becomes more attractive. Recently, increasing applications of deep neural networks (DNNs) have been reported, especially in the speech recognition and computer vision fields [21,22,23,24,25,26,27,28,29]. As a popular DNN, the deep brief network (DBN) comprises multiple layers for representing data with multilevel abstraction [22]. To describe the important trends in a combustion process, a multilayer DBN was constructed to obtain the nonlinear relationship between the flame images and the outlet oxygen content [25]. An ensemble deep kernel learning model was proposed for the melt index prediction and exhibited good predictions in an industrial polymerization process [26]. The process modeling results indicate that DNNs characterize nonlinear features better and enhance the automation level of industrial manufacturing processes. However, to the best of our knowledge, DNNs have never been applied to rubber mixing processes, especially for the Mooney viscosity modeling and prediction.

Another common challenge for a practice soft sensor development is its reliability. This is mainly because the modeling dataset often contains various outliers caused by instrument degradation, process disturbances, transmission problems, etc. [4,30,31,32]. Robust data mining approaches are necessary and more attractive for development of a reliable soft sensor in industrial processes [33,34]. A soft sensor corrupted by fitting those unwanted outliers inevitably results in erroneous predictions of the output variables. Even with some outlier detection methods as preprocessing, those inconspicuous outliers are difficult to be detected because they are masked by adjacent outliers [31]. In practice, it is more promising to develop a unified soft sensor integrated with a definite reduction of the negative effects of outliers.

To address the two above-mentioned issues simultaneously, this work aims to develop a robust DNN soft sensor for the modeling of nonlinear processes with outliers. Specially, the proposed ensemble deep correntropy kernel regression (EDCKR) framework integrates the ensemble learning [35], DBN structure [22], and correntropy kernel regression (CKR) [31,32]. The DBN-based unsupervised learning is adopted as a multilevel nonlinear feature extractor to absorb the information in related input variables. Sequentially, a supervised CKR-based prediction model is built to capture the relationship between the extracted features and the Mooney viscosity values. Without cumbersome preprocessing steps, the negative effects of outliers is reduced straightforwardly using the CKR-based robust nonlinear estimator [31]. Furthermore, with the help of ensemble learning, more reliable prediction results are obtained.

The remainder of this paper is structured as follows: In Section 2, the EDCKR-based soft sensing method with its algorithmic implemented steps is described in detail. In Section 3, its application to the Mooney viscosity prediction in an industrial rubber mixing process is presented. Finally, in Section 4 the conclusions are summarized.

## 2. Ensemble Deep Correntropy Kernel Regression Method

### 2.1. Restricted Boltzmann Machine Construction

Traditionally, using a labeled dataset $S^{l}=\left\{X^{l},Y\right\}$, supervised soft sensors are built. Different from traditional supervised learning methods, deep learning methods can integrate unsupervised and supervised learning tasks into a semi-supervised framework [21,22]. When DBN is applied to regression problems, higher-level features are learnt in the unsupervised learning stage to absorb useful information in all input data, i.e., $\left\{X^{u}\cup X^{l}\right\}$. For soft sensors, the input data are often considered as those secondary variables which can be measured online during the corresponding process. Using the extracted features, a supervised regression model is then established [25,26].

**Figure 1:**
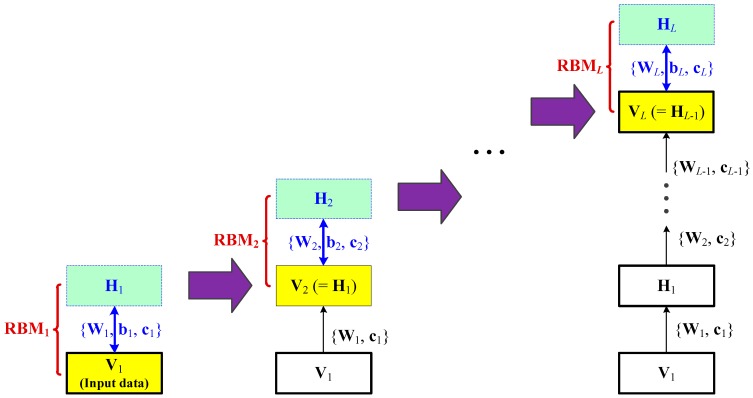
Construction of the main deep brief network (DBN) structure with multiple restricted Boltzmann machine (RBM) layers.

A brief construction of the main DBN structure with multiple layers is shown in Figure 1. With *L* individual restricted Boltzmann machine (RBM) modules represented as ${\mathrm{RBM}}_{l},l=1,\ldots ,L$, DBN can extract nonlinear features of the input data hierarchically in the unsupervised learning stage [22]. Each RBM module has a visible layer, V, related to the input data and a hidden layer, H, denoting the outputs, respectively. $V\in R^{n\times 1}$ and $H\in R^{m\times 1}$ are both vectors with binary values (one or zero). Utilizing the input data as the first visible layer $V_{1}$, the first RBM module (i.e., RBM_1_) is trained using the parameters $\theta_{1}=\{W_{1},b_{1},c_{1}\}$ to obtain $H_{1}$. With a built RBM_1_, let $V_{2}=H_{1}$, and RBM_2_ can be trained similarly. Sequentially, the RBM*_l_* module with $H_{l}$ and $V_{l}$ is trained and finally a series of RBMs are obtained [22].

The energy function E(V,H) in Equation (1) with its parameters θ={W,b,c} is utilized to describe the energy level of RBM with the available information [22].

(1) $$E\left(V,H\right)=-b^{T}V-c^{T}H-H^{T}WV$$

Specially, to construct an RBM module, the hidden layer H needs to be estimated. To achieve this aim, the probability distribution of the visual layer P(V) in Equaiton (2) is required to be maximized [22]:

(2) $$P\left(V\right)=\frac{\sum_{H}\exp\left[-E\left(V,H\right)\right]}{\sum_{V}\sum_{H}\exp\left[-E\left(V,H\right)\right]}$$

Using Equation (2), the log-likelihood function of all visible variables logM(θ) is formulated as follows:

(3) $$\begin{array}{ll} \log M\left(\theta \right) & =\sum_{v}\log P\left(V\right)\\ & =\sum_{V}\left\{\log\sum_{H}\exp\left[-E\left(V,H\right)\right]-\log\left\{\sum_{V}\sum_{H}\exp\left[-E\left(V,H\right)\right]\right\}\right\} \end{array}$$

The contrastive divergence algorithm is an effective solution to obtain the RBM structure with its parameters θ={W,b,c}. The algorithmic details can be found in [22]. Several trained RBMs are stacked sequentially to form the DBN architecture. Using the layer-by-layer feature extraction, more useful information with high-level representations is learnt from all available unlabeled data. This is helpful to further model soft sensors for quality prediction.

### 2.2. Deep Correntropy Kernel Regression Model

As aforementioned, the constructed multilayer unsupervised DBN model characterizes the input data layer-by-layer. To further train a regression model with the output data $Y={\left\{y_{i}\right\}}_{i=1}^{N}$, supervised learning methods are implemented to fine tune the weights of DBN. Therefore, the extracted features (Φ) using DBN can be suitably related to the values of the Mooney viscosity (Y). 

Recently, the kernel learning regression method and DBN were combined to construct a prediction model [26]. Compared with the traditional back propagation NN, the kernel learning regression model can be trained more easily. Additionally, it has good prediction performance, especially with limited labeled data [26]. However, the negative effects of outliers degrade the prediction performance and affect the explanation abilities. To solve this problem, using the correntropy concept [36], a supervised deep CKR (DCKR) prediction model is built to explore the relationship between the extracted features ($\Phi ={\left\{\varphi_{i}\right\}}_{i=1}^{N}$) and related Mooney viscosity values ($Y={\left\{y_{i}\right\}}_{i=1}^{N}$). Basically, the DCKR-based soft sensor model is described below [31,32].

(4) $$\begin{array}{ll} y_{i} & =f\left(\varphi_{i};\beta ,\;b\right)+e_{i}\\ & =\beta^{T}\varphi_{i}+b+e_{i},i=1,\ldots ,N \end{array}$$

where *y_i_* and *e_i_* are the process output and noise for *i*th sample, respectively; *f* is the DCKR model with its parameters β, and bias *b*, respectively.

The following optimization problem is formulated to solve the DCKR model [31,32]:

(5) $$\left\{ \begin{array}{l} \min\;J\left(\beta ,b,\rho \right)=\frac{\gamma }{2}\sum_{i=1}^{N}\rho \left(e_{i}\right)e_{i}^{2}+\frac{1}{2}{\left\|\beta \right\|}^{2}\\ s.t.\;y_{i}-\beta^{T}\phi \left(\varphi_{i}\right)-b-e_{i}=0,\;i=1,\cdots ,N \end{array}\right.$$

where the positive regularization parameter *γ* balances the model’s accuracy and complexity. Here, a simple method is adopted to select *σ* of the correntropy item $\rho \left(e_{i}\right)=\frac{\exp\left(-\frac{e_{i}^{2}}{2\sigma^{2}}\right)}{\sigma^{3}\sqrt{2\pi }}$, i.e., $\sigma =\frac{\max\left|e_{i}\right|}{2\sqrt{2}},i=1,\ldots ,N$ [31].

Using a two-level training procedure to solve the optimization problem in Equation (5) [31], the DCKR model is established in a straightforward manner. For a test input $x_{t}$, its DBN-based feature is denoted as $\varphi_{t}$. Then, the prediction ${\hat{y}}_{t}$ can be obtained.

(6) $${\hat{y}}_{t}=f(\beta ,\;b;\varphi_{t})=\sum_{i=1}^{N}\beta_{i}K\left(\varphi_{i},\varphi_{t}\right)+b$$

where $K\left(\varphi_{i},\varphi_{t}\right)$ is the kernel function of the ith sample.

The weights of samples in a trained DCKR model are $\rho \left(e_{i}\right)=\frac{\exp\left(-\frac{e_{i}^{2}}{2\sigma^{2}}\right)}{\sigma^{3}\sqrt{2\pi }},i=1,\ldots ,N$. The outliers are not expected to be fitted into the regression model. In such a situation, their fitting errors are relatively larger, and thus they have smaller weights automatically [31]. A sample is assigned with a smaller weight if it is more likely to be an outlier. Meanwhile, using a simple criterion, e.g., $\rho \left(e_{i}\right)<\bar{\rho }$ ($0.5\leq \bar{\rho }<1$ is a cutoff value after normalizing all the weights $\rho \left(e_{i}\right),i=1,\ldots ,N$ into [0,1]), the candidate outliers can be identified and removed out [32]. Interestingly, although the candidate outliers are kept in the DCKR model, they cannot degrade the prediction performance mainly because of their negligible effects. Consequently, compared with the deep kernel learning model [26], the correntropy metric-based DCKR model is more robust for outliers because it cannot amplify the outliers’ negative effects. 

It should be noticed that, in contrast to correntropy metric-based criterion, most traditional soft sensor and identification methods adopt the mean squared error loss function, which is suitable when the underlying noises obey Gaussian distribution [31,36]. However, they are sensitive to outliers. Additionally, although different weighting strategies to reduce the effect of outliers are available, most of them are not easily designed and implemented for complicated industrial data beforehand.

### 2.3. Reliability Enhancement Using Bagging-Based Ensemble Strategy

Both the quality and quantity of training data play an important role in the soft sensor model development. Unfortunately, due to the costly assaying process of the Mooney viscosity in industrial rubber mixers, the number of labeled samples is often limited. To improve the model reliability in practice, a simple bagging-based ensemble strategy [37] is integrated with the DCKR model to form EDCKR. The proposed EDCKR model generates multiple predictors and achieves an aggregated prediction.

By bootstrapped resampling the original training dataset, the bagging-based ensemble strategy generates a diversity of regression models [37]. Sequentially, the outputs are aggregated in different weighting ways [35,37,38,39]. A resampled training dataset $S_{1}^{l}=\left\{X_{1}^{l},Y_{1}\right\}$ with *N*-pair samples are randomly selected from $S^{l}=\left\{X^{l},Y\right\}$, with the probability of each pair being selected as 1N. Then *M* resampled datasets denoted as $S_{1}^{l},\ldots ,S_{M}^{l}$ can be obtained by repeating the procedure *M* times. Similarly, *M* resampled unlabeled datasets denoted as $X_{1}^{u},\ldots ,X_{M}^{u}$ are obtained. 

For $S_{m}=\left\{S_{m}^{l}\cup X_{m}^{u}\right\}$, train a DBN model to extract features $\Phi_{m}={\left\{\varphi_{m,i}\right\}}_{i=1}^{N}$. Sequentially, the corresponding DCKR model $f(\beta_{m},b_{m})$ is built using $\left\{\Phi_{m},Y_{m}\right\}$. For online prediction of $x_{t}$, its new features are denoted as $\varphi_{m,t}$. Accordingly, the DCKR-based prediction ${\hat{y}}_{m,t}$ is calculated below:

(7) $${\hat{y}}_{m,t}=f(\beta_{m},b_{m},\;\varphi_{m,t})=\sum_{i=1}^{N}\beta_{m,i}K\left(\varphi_{m,i},\varphi_{m,t}\right)+b_{m}$$

where the meanings of parameters are similar with Equation (6).

With *M* resampled datasets, altogether *M* DCKR candidate models are trained. Each DCKR candidate exhibits its individual prediction ability. Generally, a DCKR candidate with fewer outliers is more reliable. Consequently, these candidates are aggregated to a final prediction according to their reliabilities. A simple index $R_{m}$ is defined to evaluate the reliabilities.

(8) $$R_{m}=\frac{\mathrm{num}\left(\rho \left(e_{mi}\right)\geq \bar{\rho }\right)}{N}\times 100\%$$

where $\mathrm{num}\left(\rho \left(e_{mi}\right)\geq \bar{\rho }\right)$ indicates how many samples with larger weights than $\bar{\rho }$ for the *m*th DCKR candidate.

The DCKR candidate with a larger value of $R_{m}$ tends to be relatively more reliable because it is trained with fewer outliers. Consequently, the final EDCKR model for prediction is simply formulated below.

(9) $${\hat{y}}_{t}=\frac{1}{M}\sum_{m=1}^{M}\frac{R_{m}}{\sum_{m=1}^{M}R_{m}}{\hat{y}}_{m,t}$$

**Figure 2:**
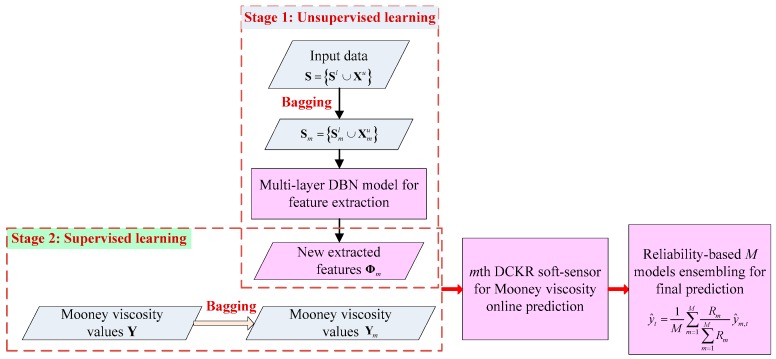
Main modeling flowchart of ensemble deep correntropy kernel regression (EDCKR) for soft sensing of the Mooney viscosity.

The main modeling flowchart of EDCKR is shown in Figure 2. Notice that all input data (i.e., those online measured secondary variables during the process) are utilized. Compared with current soft sensors for the Mooney viscosity, the EDCKR model extracts more intrinsic features using DBN and it is relatively insensitive to outliers in the modeling stage. Moreover, it is expected that, resorting to ensemble strategies, more reliable predictions can be obtained.

## 3. Industrial Mooney Viscosity Prediction

The EDCKR soft modeling approach is applied to an industrial internal mixer. Several measured variables during a short period before the discharge are chosen as secondary variables. These variables include temperature, pressure, energy, power, and duration in the mixer chamber, and they are obtainable in all batches [12,13]. They can reflect important information according to long-term accumulated process knowledge, and thus they are considered as the input data $\left\{X^{u}\cup X^{l}\right\}$. In contrast to this, the Mooney viscosity can only be assayed about every 10 batches in this manufacturing process. In such a situation, for the investigated recipe during about one month, the labeled dataset $S^{l}=\left\{X^{l},Y\right\}$ has only 140 pair of samples. Half of the labeled samples (i.e., 70 pairs) are utilized for training a model. The rest, 70 pairs, are adopted to test the prediction performance. Additionally, the unlabeled training dataset $S^{u}=\left\{X^{u}\right\}$ has about 680 input variables during the same production period in the same mixer. That is to say, for training a DCKR model, the semi-supervised training data include 680 unlabeled input samples and 70 pair labeled samples. Although obvious sampling and systematic errors can be deleted easily, the modeling dataset still has uncertainties, including process noise and those inconspicuous outliers. In this work, complex outlier defection methods are not utilized. Consequently, robust data-driven process modeling approaches are required in industrial practice.

The relative root mean squares error (RRMSE) is utilized to quantitatively evaluate the prediction performance of different soft-sensor models.

(10) $$\mathrm{RRMSE}=\sqrt{\sum_{t=1}^{N_{\mathrm{tst}}}{\left(\frac{y_{t}-{\hat{y}}_{t}}{y_{t}}\right)}^{2}/N_{\mathrm{tst}}}\times 100\left(\%\right)$$

where $y_{t}$ and ${\hat{y}}_{t}$ are the assayed and predicted values of the Mooney viscosity, respectively, for $N_{\mathrm{tst}}$ test samples.

For comparison studies, four robust soft sensors, including CKR [31,32], PCA-CKR, DCKR, and EDCKR, are investigated. Their main characteristics are described briefly in Table 1. As a supervised method, CKR shows more robustness to outliers than GPR [32]. Additionally, PCA-CKR is designed as a two-step approach by PCA-based feature extraction as preprocessing. DCKR and EDCKR are two proposed robust semi-supervised soft sensors with deep structure. The CKR, PCA-CKR, and DCKR models were trained using the cross-validation method. In particular, the value of $\bar{\rho }$ was selected as 0.8 for this case. The developed DCKR model has a five-layer structure, i.e., 14-20-10-5-1. No further constraints have been adopted in the parameter estimation stage because this is not our main aim. Additionally, for this case, relative good prediction performance of DCKR can be obtained when the number of extracted features is in the range of four to six. More features do not improve the prediction performance. Therefore, the network structure is selected by cross-validation on several candidates and the optimal is not guaranteed. 

**Figure 3:**
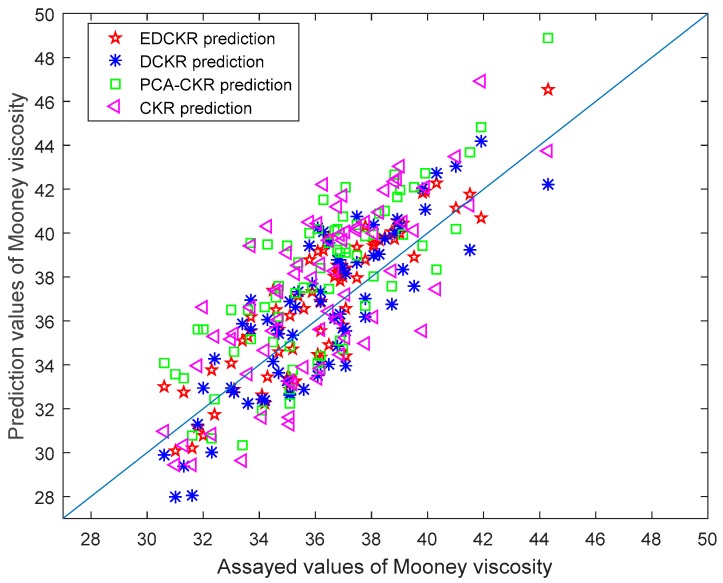
Assayed values and their prediction results of the Mooney viscosity using EDCKR, deep correntropy kernel regression (DCKR), principal component analysis and correntropy kernel regression (PCA-CKR), and correntropy kernel regression (CKR) models.

The comparisons of the Mooney viscosity prediction results are listed in Table 1. The RRMSE index indicates that EDCKR achieves the smallest prediction errors. The prediction results and their assayed values of the test data using the CKR, PCA-CKR, DCKR, and EDCKR models are shown in Figure 3. This parity plot exhibits that EDCKR and DCKR are more accurate mainly because they absorb the information of unlabeled data into a deeper structure. As shown in Table 1 and Figure 3, for feature extraction, the designed PCA-CKR model improves the prediction accuracy inapparently, inferior to DCKR. This is mainly because the two-step PCA-CKR method extracts linear features while they are not very related to sequential quality predictions.

**Figure 4:**
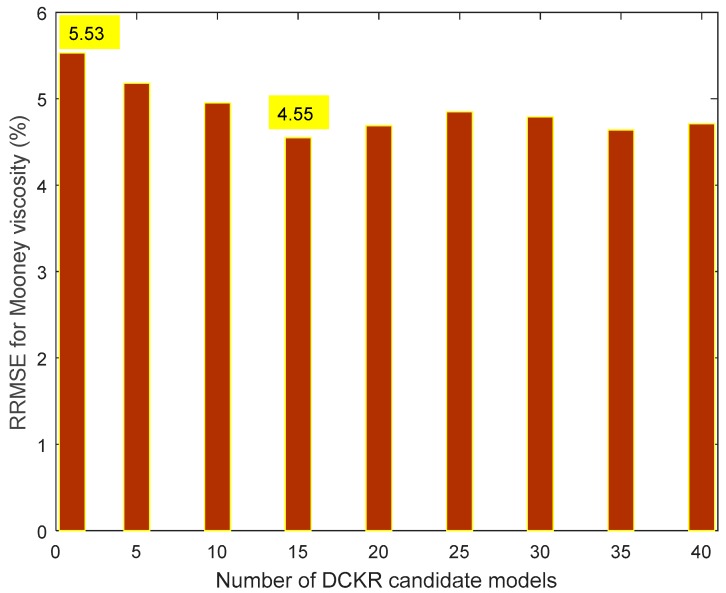
Relative root mean squares error (RRMSE) comparisons of Mooney viscosity between a single DCKR model and an EDCKR model with different candidates.

The Mooney viscosity prediction comparison results between a single DCKR model and an EDCKR one using multiple candidates are plotted in Figure 4, with different candidate numbers. Compared with a single DCKR model, the maximum improvement of EDCKR on the RRMSE index is about 1% (from 5.53% to 4.55%). As listed in Table 1, the values of maximum absolute error (i.e., $\max\left|y_{t}-{\hat{y}}_{t}\right|,t=1,\ldots ,N_{\mathrm{tst}}$) of EDCKR and DCKR methods are 3.28 and 4.16, respectively. It indicates that, compared with other methods, the reduction of maximum absolute error is obvious. Additionally, for this applied recipe, about 15 to 20 DCKR candidates are enough and more candidates are not helpful, while all the training processes are implemented offline. 

The training time required by the EDCKR is about several hours on a personal computer with a CPU main frequency of 2.5 GHz and 8 GB RAM. This is much more than that of CKR and PCA-CKR models (both of which only need several minutes). However, the model training step can be implemented offline. Using the constructed EDCKR model, the online prediction time for a test sample needs about one second. Additionally, recent deep learning training modules are available to make the training process more efficient. In practice, more importantly, the prediction performance of EDCKR is much better than that of both CKR and PCA-CKR. 

In summary, the Mooney viscosity prediction results indicate that both EDCKR and DCKR are robust semi-supervised modeling approaches, while the former is more reliable in practice. One main advantage of the recommended EDCKR method is that it can provide more accurate prediction results while the training dataset still contains noises and outliers.

## 4. Conclusions

A correntropy-based robust semi-supervised soft sensing method has been developed to predict the rubber-mixing Mooney viscosity. The proposed EDCKR-based soft sensor extracts informative features and sequentially constructs a robust prediction model without cumbersome preprocessing steps. The application results indicate that robust deep learning models are alternative tools for industrial data analytics. When new labeled and unlabeled samples are available, how to update the EDCKR model efficiently rather than training from scratch is interesting and needs to be investigated. Additionally, modeling of multiple recipes with uneven datasets, especially for those recipes with extremely limited labeled data, is a practical topic.

## Figures and Tables

**Table 1:** Comparison of the Mooney viscosity soft-sensor models: Main characteristics and prediction results.

Mooney Viscosity Soft Sensor	Main Characteristics		RRMSE (%)	Maximum Absolute Error
	Model Structure	Feature Extraction		
EDCKR (proposed)	deep (multiple)	nonlinear	4.55	3.28
DCKR (proposed)	deep	nonlinear	5.53	4.16
PCA-CKR	shallow	linear	7.71	5.86
CKR [32]	shallow	no	8.10	5.99
